# A Clinical Evaluation of Calcium and Fluoride Supplementation for Tinnitus in Non-Surgical Otosclerosis: Insights from a Tertiary Care Center in Romania

**DOI:** 10.3390/medicina61040569

**Published:** 2025-03-23

**Authors:** Andrei Osman, Alice Elena Ghenea, Ovidiu Mircea Zlatian, Lidia Boldeanu, Irina Enache, Madalina Gabriela Georgescu, Carmen Aurelia Mogoanta

**Affiliations:** 1 Department of Anatomy and Embriology, University of Medicine and Pharmacy of Craiova, 200349 Craiova, Romania; andrei.osman@umfcv.ro (A.O.); irina.enache@umfcv.ro (I.E.); 2 Otorhinolaringology Department, Emergency County Hospital of Craiova, 200642 Craiova, Romania; carmen.mogoanta@umfcv.ro; 3 Department of Microbiology, University of Medicine and Pharmacy of Craiova, 200349 Craiova, Romania; alice.ghenea@umfcv.ro (A.E.G.); ovidiu.zlatian@umfcv.ro (O.M.Z.); lidia.boldeanu@umfcv.ro (L.B.); 4 Clinical Laboratory, Emergency County Hospital of Craiova, 200642 Craiova, Romania; 5 Department of General Surgery and Qualified Care in Surgical Specialties, Carol Davila University of Medicine and Pharmacy, 050474 Bucharest, Romania; 6 Department of Otorhinolaryngology, University of Medicine and Pharmacy of Craiova, 200349 Craiova, Romania

**Keywords:** otosclerosis, tinnitus, calcium and fluoride, Tinnitus Handicap Index

## Abstract

*Background and Objectives*: The management of chronic tinnitus in patients with otosclerosis presents a considerable clinical challenge, particularly as to those who are either ineligible for or reluctant to undergo surgical interventions. Surgical interventions improve hearing levels and may provide relief from tinnitus; however, medical research is also focused on alternative non-surgical treatments aimed at symptomatic improvement. This is particularly relevant, considering that otosclerosis currently has no definitive cure, despite the existence of various surgical techniques and oral therapies. This study evaluates the effects of oral calcium and fluoride supplementation on tinnitus severity in otosclerosis patients who opted for non-surgical management. *Materials and Methods*: A total of 128 otosclerosis patients with tinnitus were included in this study, which was conducted over a five-year period. Patients were categorized into three groups based on the severity of their tinnitus (mild, moderate and severe), as assessed by the Tinnitus Handicap Inventory (THI). Patients in all three groups received Florical (Mericon Industries, Inc., Peoria, IL, USA), a calcium and fluoride supplement, and were monitored over three months. The severity of tinnitus was reassessed following supplementation. Statistical analyses were conducted to further investigate patient scores. *Results*: Evaluating patients based on the severity of their tinnitus, we consistently observed clinically significant reductions in THI scores, specifically a decrease of 10 points or more, among those with mild tinnitus. The moderate group exhibited a lower degree of reduction in their THI scores, while the severe group appeared to be unaffected. Statistical analyses reveal a significant correlation between the reduction of tinnitus and the supplementation of Florical, as the severity of tinnitus decreases. *Conclusions*: The present study suggests that oral calcium and fluoride supplementation may serve as a promising non-surgical approach for tinnitus management in otosclerosis (particularly in patients with mild symptoms), in addition to its marketing-indicated role, supporting the preservation of hearing levels in otosclerosis. Its efficacy seems to diminish as tinnitus severity increases, further pointing out a potential preventative role of this supplementation.

## 1. Introduction

Otosclerosis was first described in the 19th century [1], and to date, there is no definitive treatment available, whether surgical or pharmacological. The term ‘otosclerosis’, also known as ‘otospongiosis’, is used to describe a primary disease of the temporal bone, more precisely, one located in the bony capsule of the labyrinth. It is characterized by alternating phases of resorption and formation of bone tissue, a process led by both osteoclasts and osteoblasts [2,3] found in one or several foci in different parts of the bony capsule of the labyrinth.

Otosclerosis is a complex condition that gradually causes hearing loss. While its exact cause remains unclear, multiple theories have been proposed to explain its pathogenesis [4]. The main symptom of otosclerosis is conductive hearing loss, which occurs most frequently between the third and fifth decade of life. Out of all the patients who will develop conductive hearing loss, a proportion of 10% will also develop sensorineural hearing loss [5]. In addition, 56% to 84.5% of patients with otosclerosis experience tinnitus, especially in cases of cochlear involvement and progressive hearing loss [6]. Currently, there are no reliable treatment protocols for addressing chronic tinnitus, as therapy is difficult to adjust due to the fluctuating nature of tinnitus, as well as its association with hearing loss, its multiple causes and the widespread variability in defining and reporting the results of relevant studies [7].

Managing hearing loss and tinnitus and improving the quality of life in patients with otosclerosis presents a significant clinical challenge, as recognized by many otologists and audiologists. Over the years, various therapeutic strategies have been investigated, with recent studies continuing to contribute to this area of research. Surgical intervention, particularly stapedectomy, has demonstrated efficacy in reducing tinnitus symptoms associated with otosclerosis [8], in addition to improving hearing levels. However, the long-term success of such interventions varies among individuals, particularly due to the availability of multiple surgical techniques [9] and life-long disease progression. Surgical interventions alone (whether stapedectomy or stapedotomy) are useful for improving hearing levels but seem only partially efficient in improving chronic tinnitus [6,8,9].

The challenge is especially significant in patients who are either ineligible for or choose not to undergo surgery. Considering the available research, a multidisciplinary approach combining both surgical and non-surgical modalities appears to offer the most promising results in managing tinnitus in otosclerosis patients [8,10,11], with clinical practitioners needing constant adjustment of their management plans in order to improve patients’ quality of life. Similar to surgical therapies, non-surgical approaches to managing otosclerosis also address the preservation of hearing levels, and their impacts on tinnitus are still being investigated [11]. Consequently, further research is required to establish standardized treatment protocols for tinnitus management in this target population group.

## 2. Materials and Methods

In this observational, prospective single-arm study, we aimed to evaluate changes in tinnitus severity in otosclerosis patients who opted not to undergo surgical treatment and opted for supportive oral therapy. The current study was conducted between May 2020 and March 2024 within our hospital in Craiova, Romania. Data were collected over the 5-year period from 128 patients diagnosed with otosclerosis in the Otorhinolaryngology Department at the Clinical Emergency County Hospital in Craiova.

Patients in our study received oral calcium and fluoride supplements as a singular therapy, and tinnitus severity was monitored using the Tinnitus Handicap Inventory (THI) [12]. The selected supplement formulation was Florical (Mericon Industries, Inc., Peoria, IL, USA). Florical consists of capsules containing 3.75 mg of sodium fluoride and 145 mg of calcium carbonate as the active ingredients. The inactive ingredients included microcrystalline cellulose (a commonly utilized filler and binder that maintains the structural integrity of the capsule), magnesium stearate (an anti-caking agent designed to prevent the ingredients from adhering during manufacturing), silicon dioxide (a flow agent used to enhance the consistency of the powder and prevent clumping) and gelatin. (The capsule shell is composed of gelatin, facilitating the oral delivery of the active ingredients, and is typically sourced from animal products.) Patients enrolled in the study were instructed to take three capsules daily, one at the start of each meal.

Because we chose to start supplementation at a higher dose, we also monitored patient safety and potential adverse effects of the calcium and fluoride supplementation. With these goals in mind, several blood tests were performed prior to study inclusion. Liver function was assessed by measuring hepatic enzymes, including aspartate aminotransferase (AST), alanine aminotransferase (ALT), alkaline phosphatase (ALKP) and acid phosphatase (ACP), as elevated levels of these enzymes may indicate fluoride-induced liver toxicity [13]. Additionally, serum fluoride levels were measured to evaluate prior fluoride exposure and potential toxicity [14]. To exclude any kidney dysfunction that would contraindicate further administration of calcium and fluoride, hemoglobin, blood urea and creatinine levels were investigated [6]. Hemoglobin levels were assessed to exclude anemia. Additionally, patients were questioned about any history of pain or swelling in the joints, renal disorders, and calcium oxalate kidney stones, as well as any signs of synovitis or plantar fasciitis [15].

The diagnosis of otosclerosis in patients was established through a comprehensive assessment utilizing ear (otic) endoscopy (to rule out any tympanic membrane pathology), pure tone audiometry, impedance tympanometry and stapedial reflex testing [16]. Transient evoked otoacoustic emissions [16] were used for differential diagnosis in cases that were clinically ambiguous or presented diagnostic uncertainty.

The study cohort was selected by consecutive sampling in order to minimize selection bias. Out of a total number of 252 otosclerosis patients diagnosed within our medical facility during these 5 years, 176 patients opted not to undergo surgical treatment. Of these, 128 were included in the present study, and treated for tinnitus using only oral calcium and fluoride supplementation. The remaining 48 patients were excluded from the study, either due to the absence of tinnitus symptoms, ongoing liver disease or anemia, cardiovascular disease (with or without chronic medication), chronic kidney disease, or their refusal to participate. These exclusion criteria helped minimize potential bias confounders that could separately affect tinnitus severity. The patient’s consent to participation in the study was obtained for each individual and attached to their patient files on the date of diagnosis.

Upon inclusion in the study, a computed tomography (CT) scan of the temporal bone [17,18] was conducted to accurately identify the typical otosclerosis lesions within the labyrinthine capsule, middle ear and cochlear endosteum.

Male and female patients were included in the study cohort based on the following inclusion criteria:

- Age ≥18 years (age in our cohort ranged from 20 to 63 years old);
- Diagnosis of otosclerosis confirmed through audiometric evaluation and clinical examination (normal tympanic membranes, a history of conductive hearing loss with a bilateral type A tympanogram, bilaterally absent stapedial reflex [16]);
- A CT scan indicating the typical hypodense lesions of the bony capsule of the labyrinth;
- Subjective chronic presence of tinnitus;
- No other ongoing hearing interventions;
- Provided written informed consent to participate in the study.

Exclusion criteria were based on the following:

- Patient refusal to participate;
- Absence of tinnitus symptoms, despite having a confirmed diagnosis of otosclerosis;
- Presence of ongoing liver disease or anemia, conditions that could interfere with calcium and fluoride metabolism;
- Cardiovascular disease (with or without chronic medication), due to its potential impact on tinnitus;
- Chronic kidney disease;
- Patients with psychiatric comorbidity.

The final step prior to the start of supplementation involved completing the Tinnitus Handicap Inventory (THI) questionnaire and assessing tinnitus severity using a grading scale established by previous research [12].

These patients were monitored over a three-month period, after which their tinnitus symptoms and treatment responses were systematically reassessed. During this period, hearing levels were not reassessed unless patients presented with new complaints. Significant adverse reactions, such as skin rashes, recurrent vomiting, unexplained fatigue [14] (indicative of potential anemia), and painful or tender joints, were closely monitored through monthly patient check-ins. The three-month evaluation consisted of repeating the pure tone audiometry, conducting the full bloodwork panel, and having patients retake the Tinnitus Handicap Inventory (THI). Patients who demonstrated a reduction in their total THI score were instructed to continue calcium and fluoride supplementation at a reduced dosage. In contrast, patients with no improvement in their scores were discontinued from the supplements and underwent a multidisciplinary reevaluation [19] to determine an alternative course of therapy. For patients who opted to continue the supplementation after the study was concluded, the recommended dose was one capsule per day.

After obtaining the final THI scores, statistical analyses were conducted to determine whether the supplementation led to significant reductions in THI scores and whether these reductions varied across the severity groups. The dataset included 127 patients, categorized into three groups: the ‘mild tinnitus’ group (total *n* = 41), the ‘moderate tinnitus’ group (*n* = 59) and the ‘severe tinnitus’ group (*n* = 27). The primary outcome variable, THI score variation (named ‘thi_diff’), was calculated as the difference between the THI score after supplementation and the THI score before supplementation. Negative values indicate an improvement in tinnitus severity. The dataset included one patient not included in the statistical analysis.

We used descriptive statistics like the mean and standard deviation, the Shapiro–Wilk normality test, group comparison tests (paired Student’s *t*-test, One-Way ANOVA—Analysis of Variance, and Post Hoc Comparisons—Bonferroni Test). We also employed proportions and frequency analysis and utilized boxplots as a data visualization technique.

Based on the existing literature, we chose to consider the clinical improvement of tinnitus as a decrease of 10 points or more in the THI scores of patients [20,21,22]. THI score variations of less than 10 points were classified as mild improvement or mild worsening and were attributed to natural tinnitus fluctuations, the effect of supplementation, or a combination of both. An increase in score of 10 points or more would also be considered a clinically significant worsening of tinnitus. Based on our established 10-level score improvement grading, a binary variable, named ‘significant_thi’, was generated to classify cases where THI scores decreased by more than 9 points, indicating a clinically significant reduction in tinnitus severity.

## 3. Results

### 3.1. THI Score Results and Variation

The study cohort involves 128 patients diagnosed with otosclerosis, all of whom opted for conservative management of otosclerosis instead of undergoing surgical intervention. The cohort patients presented with either conductive or mixed hearing loss, with varying degrees of severity, ranging from mild to moderate (both first and second degrees of moderate hearing loss) [23]. The THI scores obtained, which provide a measure of tinnitus-related disability, were categorized into mild (score 18–36), moderate (score 38–56) and severe (score 58–76) levels, according to pre-existent recommendations [12,20]. After the three months of supplementation, some patients transitioned into the ‘very mild’ category of tinnitus (score 0–16). Before oral supplementation, 41 patients had mild THI scores and were included in the ‘mild tinnitus’ group. Mild tinnitus is classified in the THI as occasionally interfering with sleep but not with daily activities. Another 60 patients reported a moderate THI score, and were included in the ‘moderate tinnitus’ group. Moderate tinnitus is more pronounced and is perceived even in the presence of environmental sound; the interference with sleep and relaxing activities is not infrequent. The remaining 27 patients exhibited severe THI scores and were included in the ‘severe tinnitus’ group. This reflects a significant handicap due to tinnitus. It is continuously perceived and hardly masked by external noise. Most of these patients complained of an altered sleep cycle and relaxing activities being compromised. No patients registered a ‘very mild’ score at baseline evaluation. The ‘very mild’ category of tinnitus is defined by THI pre-existing data as being perceived only in silence and easily masked [12]. It does not interfere with sleep or with daily activities and it is the lowest score classifiable by the THI.

These initial assessments served as the baseline for evaluating the efficacy of the calcium and fluoride supplementation over the following three months.

**Figure 1:**
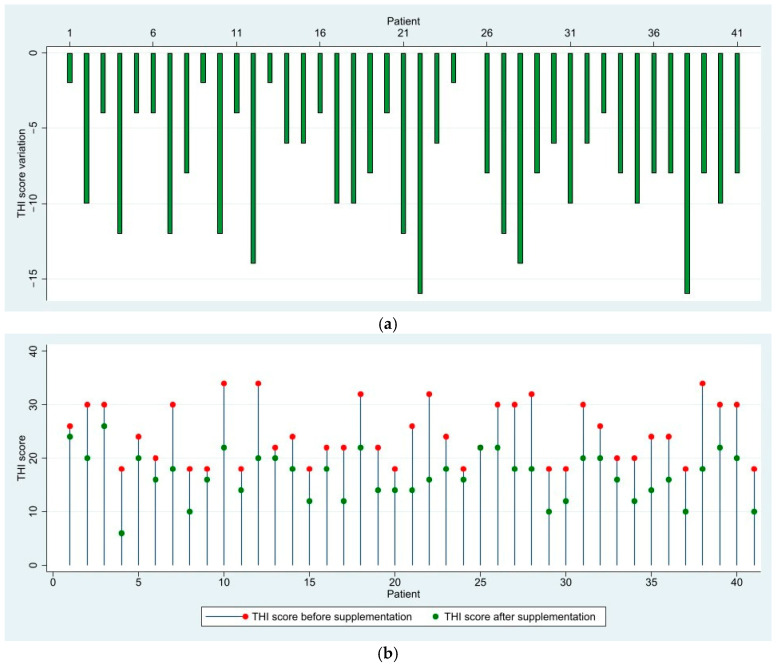
(**a**) Variation in THI scores after 3 months of supplementation—Mild Group; (**b**) Visualization of THI scores before and after supplementation—Mild Group.

After three months of oral supplementation, in the mild group, which consisted of 41 patients, 15 patients (36.59%) achieved a clinically significant reduction in their THI score and 25 patients (60.97%) displayed mildly improved scores. Only one patient showed no change in their score, and no patients reported an increased THI score. Overall, 40 out of 41 patients (97.56%) experienced a decrease in THI scores, confirming a positive effect on tinnitus. In total, 13 patients transitioned to the ‘very mild’ category of tinnitus after oral supplementation. No patients in this group displayed any changes in their audiometry-measured hearing thresholds, but it is worth noting that all patients in the group had mild and first degree moderate conductive hearing loss (Figure 1).

**Figure 2:**
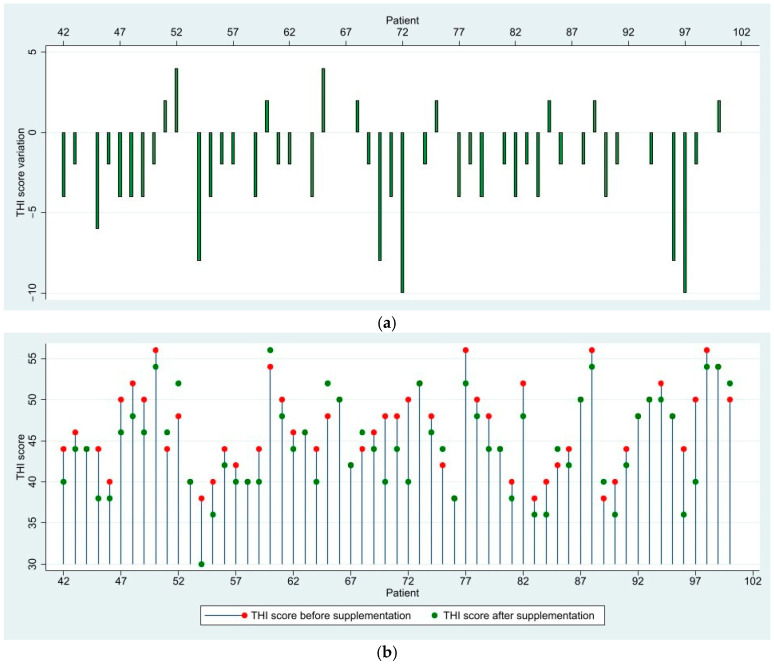
(**a**) Variation in THI scores after 3 months of supplementation—Moderate Group; (**b**) Visualization of THI scores before and after supplementation—Moderate Group.

In the moderate group, consisting of sixty patients, only two patients (3.39%) reached the clinically significant reduction threshold in their THI scores. In total, thirty-four patients (57.63%) showed mild improvement in their scores; fourteen patients (23.73%) remained completely stationary, with no change in THI scores; and nine patients (15.25%) showed a small increase in THI scores, but without exceeding 10 points. One female patient was removed from the study and was advised to discontinue the use of Florical after describing tender elbow and shoulder joints. She was lost to follow-up and excluded from the analysis. Six patients transitioned into the mild category of tinnitus, and one patient into the severe category. While the overall response to supplementation was positive, this group had lower decreases in scores and more patients with stationary or heightened scores compared to the mild group. All patients in this group had moderate conductive or mixed hearing loss (first or second degree) and the hearing thresholds were not affected during the three months of supplementation (Figure 2).

**Figure 3:**
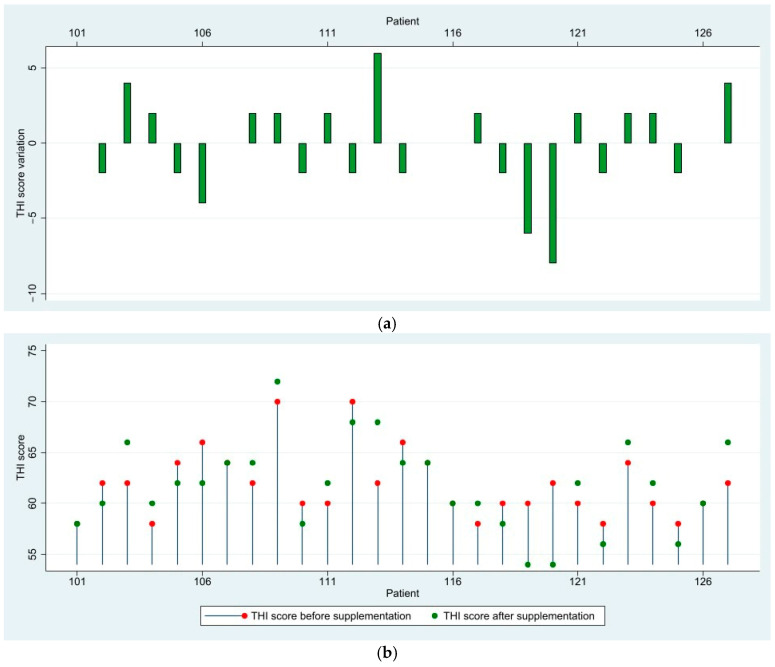
(**a**) Variation in THI scores after 3 months of supplementation—Severe Group; (**b**) Visualization of THI scores before and after supplementation—Severe Group.

In the severe group, which included 27 patients, no patients experienced a clinically significant reduction or increase in their THI scores. A subset of 6 patients (22.22%) remained completely stationary. The severe group exhibited the least favorable response to treatment, and this was reflected in the low variation of the scores. Eleven patients (40.74%) showed mild improvement in THI scores. Ten patients (37.04%) experienced worsening of THI scores, though none exceeded the 10-point threshold. After supplementation, four patients transitioned from severe tinnitus to moderate, with THI scores of 56 and 54. Patients in this group had second degree mixed moderate hearing loss and some of the patients with increasing scores displayed a small increase of 5 to 10 dB in their air-conduction thresholds at 4 and 8 kHz (Figure 3).

Patients included in the study did not experience uncomfortable side effects from the high doses of Florical, except for one female patient from the ‘moderate tinnitus’ group who complained of tender and swollen joints and was removed from supplementation. Liver and kidney tests during the supplementation showed a steady increase in monitored serum enzyme levels (AST, ALT, ALKP and ACP) but these did not reach values outside of normal ranges. We did not register any patients who developed anemia or other types of toxicity.

Detailed THI scores, are available in the accompanying Supplementary Materials, as follows: THI scores for the ‘mild tinnitus’ group may be found in Workbook1, THI scores for the ‘moderate tinnitus’ group may be found in Workbook2 and THI scores for the ‘severe tinnitus’ group may be found in Workbook3.

### 3.2. Statistical Analysis of THI Scores

#### 3.2.1. Descriptive Statistics for the THI Score Variation

The mean and standard deviation of THI score changes were computed for each group and analyzed (Table 1). The ‘mild tinnitus’ group showed the greatest reduction in THI scores, while the ‘severe tinnitus’ group exhibited minimal improvement. Mild improvement can also be seen in the ‘moderate tinnitus’ group.

#### 3.2.2. Normality Test (Shapiro–Wilk)

Shapiro–Wilk tests confirmed that THI score variations in each group followed a normal distribution (*p* > 0.05 for all groups), justifying the use of parametric tests.

#### 3.2.3. One-Way ANOVA and Post Hoc Comparisons

A one-way ANOVA was conducted to compare THI score variations among groups:

(1) F(2,124) = 51.52, *p* < 0.0001, indicating a statistically significant difference in THI reductions across severity groups.
(2) Post hoc Bonferroni tests revealed significant differences in some groups;
  (a) Mild vs. moderate tinnitus: *p* < 0.0001 (significant difference);
  (b) Mild vs. severe tinnitus: *p* < 0.0001 (significant difference);
  (c) Moderate vs. severe tinnitus: *p* = 0.077 (not statistically significant).

#### 3.2.4. Proportion of Patients with Significant THI Reduction

The proportions of clinically significant THI score reductions are displayed in Table 2. A clinically significant reduction is seen in the ‘mild tinnitus’ group and can be identified to a lesser extent within the ‘moderate tinnitus’ group as well. The ‘severe tinnitus’ group did not exhibit a clinically significant reduction in their scores.

**Figure 4:**
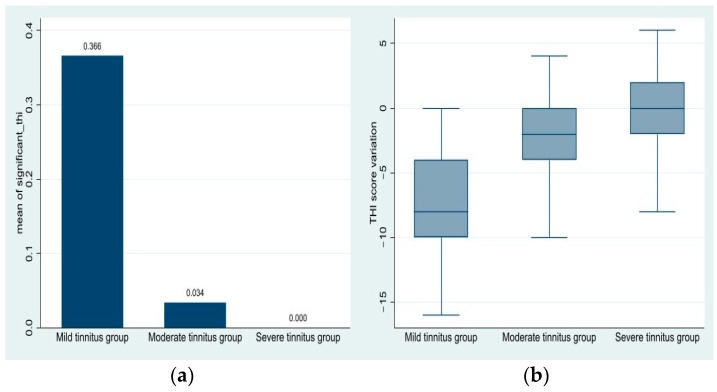
(**a**) Means of the binary variable ‘significant_thi’ for each group; (**b**) THI score variation per group.

These findings indicate that supplementation efficacy decreases as tinnitus severity increases, as visualized in Figure 4. Patients in the ‘mild tinnitus’ group experienced significantly greater reductions in THI scores than those in moderate or severe groups. The results, besides the clinical improvement stated in the questionnaires, are supported by determinations of statistical significance in the ANOVA and post hoc comparisons, reinforcing the idea that greater initial tinnitus severity correlates with lessened responses to Florical administration. Florical seems to have a good impact on tinnitus in patients with lower tinnitus scores.

## 4. Discussion

This study evaluated the impact of calcium and fluoride supplementation (Florical) on tinnitus severity in 128 patients with otosclerosis who opted for conservative management of their condition instead of surgery. After three months of supplementation, the patients with a mild degree of tinnitus experienced a significant good response, with noticeable reduction of tinnitus severity as measured by THI. The patients with moderate tinnitus, measured by the same means, experienced a lesser improvement, while the patients with severe tinnitus seemed unaffected.

The THI is a widely used tool used to assess the impact of tinnitus on patients’ lives, including patients diagnosed with otosclerosis, especially since tinnitus is a common symptom in otosclerosis, affecting 65–90% of patients and often co-occurring with hearing loss [24]. For our given topic, we chose THI in order to identify specific complaints and report the severity of tinnitus symptoms in our cohort of patients. The literature indicates that otosclerosis patients often report a range of issues using the THI, with certain items being more frequently highlighted as problematic [25]. Emotional distress as an aspect of tinnitus, such as reports of frustration and irritability (“Does your tinnitus make you feel anxious?”; “Does your tinnitus make you feel frustrated?”; “Does your tinnitus make you feel depressed?”), was frequently and highly reported (4 points) by our patients. The emotional subscale of the THI, which includes items related to feelings of depression and anxiety, is often highlighted as a significant area of concern in specific studies [25]. In our study, the emotional distress factor consistently scored high grades for all patients, and some downgrades were recorded in patients who had lowering scores. The above-mentioned questions were often graded with the maximum score in our study and were consistently validated in all patients with high scores.

Social function impairment is present in many tinnitus patients and most often in otosclerosis patients with ‘severe’ scores on the THI [25]. Our reports on difficulties in concentration and sleep (“Does your tinnitus make it difficult for you to concentrate?”; “Does your tinnitus make it difficult to sleep?”; “Do you find it difficult to focus on a task because of tinnitus?”) showed significant impacts on patients in all groups. They were the problems most consistently reported with high scores across patients in all our study groups. The lowering of these scores was a consistent finding in all patients with decreasing tinnitus symptoms, whether clinically relevant or considered a physiological fluctuation of tinnitus. The functional domain of THI seems most affected in otosclerosis patients [25], as studies indicate, and was the most consistently improved domain in all our patient groups following Florical administration.

Catastrophic reactions linked to THI items (“Do you feel as though you cannot escape your tinnitus?”; “Because of your tinnitus, do you feel that you have a terrible disease?”), such as fear of severe illness or inability to cope with tinnitus, were not a common report in our study, mostly being graded with 0 points. The current literature suggests that these items report directly on the severe and catastrophic impacts of tinnitus, and its connection to the mental health and overall well-being of the patient [24]. In our study, patients who did respond to these questions with at least two points saw increasing levels in tinnitus as measured with the THI after the three months. Only a small subset of otosclerosis patients seem to have catastrophic tinnitus manifestations, and in these cases, the etiology of catastrophic symptoms is difficult to attribute to only one factor alone, and no matter the chosen treatment plan, results seem poor [26].

The worsening of tinnitus in otosclerosis patients cannot be solely attributed to anxiety, as the relationship between the two may suggest. While anxiety is a significant factor, it is not the only one influencing tinnitus severity. Certain studies [27] suggest that hearing loss in otosclerosis is the primary driver of tinnitus, rather than anxiety alone. Various studies have explored the correlation between anxiety and tinnitus, but the evidence does not support a direct and unique causal relationship [28]. Instead, tinnitus severity in otosclerosis patients is likely influenced by a combination of factors. A Mendelian randomization study found no genetic causality between anxiety and tinnitus severity, suggesting that while anxiety and tinnitus are correlated, anxiety alone does not cause tinnitus to worsen [29,30]. During the COVID-19 pandemic, increased anxiety levels were observed, but this did not correspond to a significant increase in tinnitus severity in patients, further suggesting that anxiety alone does not dictate tinnitus outcomes [31].

In patients with otosclerosis and tinnitus, the relationship between worsening tinnitus symptoms and lowered air-conduction thresholds at 4 and 8 kHz is complex and may have heterogeneous explanations. Some of our patients with increasing THI scores and worsening tinnitus displayed lowered air-conduction thresholds at the mentioned frequencies. The progression of otosclerosis can affect hearing thresholds, and surgical interventions like stapedotomy are commonly used to improve hearing levels and alleviate tinnitus [8]. However, the specific impact on high-frequency air-conduction thresholds, such as those at 4 and 8 kHz, requires careful consideration. Studies indicate that otosclerosis leads to elevated air-conduction thresholds, particularly in its advanced stages. Early otosclerosis shows higher air-conduction thresholds compared to controls, and these thresholds are significantly higher in late-stage otosclerosis [32]. Stapedotomy is shown to significantly improve air-conduction thresholds and reduce the air–bone gap, which can lead to improved hearing outcomes [33]. However, the impact on high-frequency thresholds like 4 and 8 kHz is not consistently detailed across multiple studies. While the focus is often on monitoring speech frequencies, high-frequency hearing can also be affected. Postoperative improvements in air-conduction thresholds are noted, but specific data in 4 and 8 kHz improvement or degrading of thresholds are less frequently reported [34,35]. In the absence of any surgical intervention and other tinnitus medication, the three-month period in our study seems to have been not enough time for the lowering of hearing thresholds dure to otosclerosis progression. The severity of tinnitus may be to blame for the lowered air-conduction levels, but a direct link may not be fully credible. Even if increasing tinnitus may indicate a correlation with high-frequency threshold degradation, the link is not well-documented [24,36] and should be further investigated.

When a patient with otosclerosis experiences an improvement in tinnitus symptoms under a specific treatment, it can be considered significant, especially in the absence of other tinnitus medications, a fact taken into account by our study. This significance is underlined by the fact that chronic tinnitus is considered multifactorial and a challenging symptom to address in general [37]; a multitude of treatments are available [38] and still being tested, speaking to the unyielding nature of this life-long symptom. Otosclerosis surgery is still a popular treatment option for patients, with studies indicating that a substantial percentage of patients report a reduction or complete cessation of tinnitus post-surgery. For instance, one study found that 82.8% of patients experienced a complete or partial absence of tinnitus one month after surgery [35]. Another study reported that 89% of patients had cessation of tinnitus one year post-surgery [36]. Conservative treatments with sodium fluoride and bisphosphonates have been shown to stabilize hearing thresholds and delay the worsening of tinnitus. These treatments are particularly beneficial in the early stages of otosclerosis [11,39]. Bisphosphonates are currently showing a promising effect as a conservative tinnitus management in otosclerosis patients [40], with some patients registering complete tinnitus resolution at six months. While studies show good efficiency, even in patients with residual post-surgery tinnitus, it is worth noting that risedronate (bisphosphonates) has more systemic risks, requiring closer monitoring [40] for adverse effects.

Our findings suggest that calcium and fluoride supplementation may provide significant relief for patients with mild otosclerosis-associated tinnitus, a new approach that is currently less explored in clinical practice. Unlike the traditional management strategies that focus on surgery or symptomatic relief through cognitive approaches [41], our study introduces a potential biochemical calcium-dependent pathway for tinnitus modulation in otosclerosis, one that other studies have explored [42]. This suggests that tinnitus severity could be linked to calcium metabolism [43] and cochlear homeostasis, a finding offering new insights into disease progression and intervention timing.

The significant improvement which we observed in mild tinnitus cases suggests that early supplementation might alter the disease course before tinnitus worsens. However, as tinnitus severity increases, the response to supplementation diminishes, raising important questions about the optimal timing and patient selection for this potential therapy. Our observational study highlights a possible new alternative in tinnitus management and may open the way for future randomized clinical controlled trials (RCCT) to validate calcium and fluoride as a treatment approach or potential preventive strategy in otosclerosis-related tinnitus.

The three-month follow-up period we selected was based on prior research indicating that effective tinnitus therapies should typically produce measurable effects within this timeframe [44,45]. The rather short period is, however, a key limitation of our study. While this period of time has allowed us to assess the initial effects of calcium and fluoride supplementation on tinnitus severity, it does not provide insight into the long-term stability of these improvements, despite most patients having returned annually for audiometric check-ups with stable results. Alongside RCCT, future studies with extended follow-up periods would be necessary to further evaluate treatment effects and potential relapses before a formal validation of this supplementation as standardized therapy.

## 5. Conclusions

Our study provides preliminary evidence that oral calcium and fluoride supplementation may be an effective non-surgical option for managing tinnitus in otosclerosis patients, particularly those with mild tinnitus. The significant reductions in THI scores observed in the ‘mild tinnitus’ group suggest that supplementation could play a safe role in early-stage symptom management. Given the existing pharmacological approaches such as sodium fluoride [11,39], bisphosphonates [40], or cognitive therapy [41], our current findings highlight a potential complementary or preventive role for calcium and fluoride in tinnitus management.

In addition to RCCT, further research could focus on elucidating the mechanisms by which calcium and fluoride influence tinnitus perception and otosclerosis progression, as well as assessing the long-term sustainability of these effects. Additionally, investigating potential benefits in post-surgical otosclerosis patients may offer valuable information as to whether supplementation could serve as a complementary treatment for patients who have undergone stapedotomy and yet still experience tinnitus post-surgery.

## Figures and Tables

**Table 1:** The means and standard deviations of THI score variations for all groups.

			Mild Tinnitus *n* = 41		Moderate Tinnitus *n* = 59		Severe Tinnitus *n* = 27		Total *n* = 127	
THI score Mean ± SD (min-max)			Baseline	3 months	Baseline	3 months	Baseline	3 months	Baseline	3 months
			24.49 ± 5.58 (18–34)	16.73 ± 4.44 (6–26)	46.37 ± 5.04 (38–56)	44.44 ± 5.80 (30–56)	61.85 ± 2.32 (58–70)	61.70 ± 4.39 (54–72)	42.60 ± 14.72 (18–70)	39.16 ± 17.64 (6–72)
THI score difference Mean (min-max)			−7.76 (−16–0)		−1.93 (−10–4)		−0.15 (−8–6)		−3.43 (−16–6)	
*p*-value			<0.001		<0.01		0.805		<0.01	

**Table 2:** Significant THI reductions.

Group	Total Patients	% Significant Reduction
Mild tinnitus	41	36.59%
Moderate tinnitus	59	3.39%
Severe tinnitus	27	0.00%

## Data Availability

The data presented in this study are available on request from the corresponding author.

## Supplementary Materials

The following supporting information can be downloaded at: https://www.mdpi.com/article/10.3390/medicina61040569/s1. Supplementary file: Workbook 13 corresponding to Figure 1, Figure 2 and Figure 3.

## Abbreviations

The following abbreviations are used in this manuscript:

THI	Tinnitus Handicap Inventory
AST	Aspartate aminotransferase
ALT	Alanine aminotransferase
ALKP	Alkaline phosphatase
ACP	Acid phosphatase
CT	Computed tomography
RCCT	Randomized clinical control trial
